# Heritability of autism spectrum disorders: a meta‐analysis of twin studies

**DOI:** 10.1111/jcpp.12499

**Published:** 2015-12-27

**Authors:** Beata Tick, Patrick Bolton, Francesca Happé, Michael Rutter, Frühling Rijsdijk

**Affiliations:** ^1^ MRC Social, Genetic and Developmental Psychiatry CentreIOPPNKing's College LondonLondonUK

**Keywords:** Autism spectrum disorders, meta‐analysis, heritability, twin studies, DF extremes analysis

## Abstract

**Background:**

The etiology of Autism Spectrum Disorder (ASD) has been recently debated due to emerging findings on the importance of shared environmental influences. However, two recent twin studies do not support this and instead re‐affirm strong genetic effects on the liability to ASD, a finding consistent with previous reports. This study conducts a systematic review and meta‐analysis of all twin studies of ASD published to date and explores the etiology along the continuum of a quantitative measure of ASD.

**Methods:**

A PubMed Central, Science Direct, Google Scholar, Web of Knowledge structured search conducted online, to identify all twin studies on ASD published to date. Thirteen primary twin studies were identified, seven were included in the meta‐analysis by meeting Systematic Recruitment criterion; correction for selection and ascertainment strategies, and applied prevalences were assessed for these studies. In addition, a quantile DF extremes analysis was carried out on Childhood Autism Spectrum Test scores measured in a population sample of 6,413 twin pairs including affected twins.

**Results:**

The meta‐analysis correlations for monozygotic twins (MZ) were almost perfect at .98 (95% Confidence Interval, .96–.99). The dizygotic (DZ) correlation, however, was .53 (95% CI .44–.60) when ASD prevalence rate was set at 5% (in line with the Broad Phenotype of ASD) and increased to .67 (95% CI .61–.72) when applying a prevalence rate of 1%. The meta‐analytic heritability estimates were substantial: 64–91%. Shared environmental effects became significant as the prevalence rate decreased from 5–1%: 07–35%. The DF analyses show that for the most part, there is no departure from linearity in heritability.

**Conclusions:**

We demonstrate that: (a) ASD is due to strong genetic effects; (b) shared environmental effects become significant as a function of lower prevalence rate; (c) previously reported significant shared environmental influences are likely a statistical artefact of overinclusion of concordant DZ twins.

## Introduction

Autism is known as a severe pervasive neurodevelopmental disorder with poor prognosis. The disorder is characterized by difficulties in social interaction, verbal and nonverbal communication and repetitive behaviours, and is more prevalent in males (Le Couteur & Szatmari, 2015). It has a considerable impact on the family as well as social, educational and health care systems. A lot of research effort has gone into understanding the causes of individual differences in autistic behaviour, with clear evidence for genetic effects.

Twin studies of the heritability of Autism Spectrum Disorders [ASD; an umbrella term denoting autism, Asperger syndrome and Pervasive Developmental Disorder Not Otherwise Specified (NOS)] have been reviewed and summarized most recently by Ronald & Hoekstra (Ronald & Hoekstra, 2011). Their review included the seven primary studies published up to 2011, annotated in Table 1.

**Table 1:** List of primary twin studies on Autism Spectrum Disorder included and excluded in the meta‐analysis

Source	Country	Systematic recruitment; (*n* pairs)	Blind to zygosity & cotwin status; DZ OS included	Basis for diagnosis	Diagnostic criteria	Reason for exclusion	Ascertainment type
Included studies							
3. Steffenburg et al. (1989)	Nordic Regions	Yes; included triplets; (21)	No; No	Records and interview; ABC, the Lotter checklist & DIPBEC	DSM III R	Autistic Disorder^a^	CA
5. Le Couteur et al. (1996)**	United Kingdom	Yes; included triplets; (48)	No; No	Records and interview; ADI, ADOS	ICD 10 & DSM IV	Broader Phenotype^b^, Autistic Disorder^c^, Atypical Autism^d^	CA
6. Taniai et al. (2008)	Japan	Yes; (45)	Yes; Yes	Records and semistructured interview; CARS‐TV	DSM IV	Autistic Disorder^c^, Asperger Syndrome^e^, PDD‐NOS^f^	CA
8. Lichtenstein et al. (2010)	Sweden	Yes; (7982)	*n*/a; Yes	Records and telephone interview; A‐TAC	DSM IV	Pervasive Developmental Disorders^g^	RPA
9. Hallmayer et al. (2011)	United States	Yes; (192)	*n*/a; Yes	Records and interview; ADI, ADOS	DSM IV	Autistic Disorder^c^, ASD^h^	IA (*π *= .96)
12. Nordenbæk et al. (2014)	Denmark	Yes; (36)	Yes; No	Records and interview; ADOS, DISCO	ICD 10 & DSM IV	Autistic Disorder^c^, Asperger Syndrome^e^, PDD‐NOS^f^	IA (*π *= .76)
13. Colvert et al. (2015)	United Kingdom	Yes; (127)	Yes; Yes	Records and interview; CAST, DAWBA, ADI, ADOS, Best‐estimate Diagnosis	ICD 10 & DSM IV	Autism Spectrum Disorder^h^, Broad Spectrum Disorder^i^	CA
Excluded studies						Reason for exclusion	
1. Folstein and Rutter (1977)**	United Kingdom	Yes	Yes; No	Records and interview	Criteria of DSM released in 1980	Superseded by Le Couteur et al., 1996;	CA
2. Ritvo et al. (1985)*	United States	No	Not specified; Yes	Records and interview	DSM III	Biased Systematic Recruitment	IA (*π *= .86)
4. Bailey et al. (1995)**	United Kingdom	Yes; included triplets	Yes; No	Records and interview	ICD 10	Superseded by Le Couteur et al., 1996;	CA
7. Rosenberg et al. (2009)*	United States	No	No; Yes	Voluntary Registry: care‐giver reported diagnosis of ASD	DSM IV TR	Did not meet Systematic Recruitment criterion	*n*/a*
10. Frazier et al. (2014)*	United States	No	No; Yes	Voluntary Registry: care‐giver reported diagnosis of ASD	DSM IV TR	Did not meet Systematic Recruitment criterion	*n*/a*
11. Sandin et al. (2014)**	Sweden	Yes	*n*/a; Yes	National Registry Diagnostic information	ICD‐10	No concordance information, and, Lichtenstein et al. (2010) already reported on at least part of this twin sample	CA

CA = complete (or double) ascertainment (*π *= 1); IA = incomplete ascertainment (0 < *π *< 1), *π* is calculated; RPA = random population ascertainment; DSM = Diagnostic and Statistical Manual of Mental Disorders; ICD = International Classification of Diseases.

Study number is assigned chronologically based on publication year. Studies 1 and 4 (**) were excluded on the basis that they were superseded by a more recent study of the same research group; study 11 (**) did not provide twin concordance data, additionally, Lichtenstein et al. (2010) have already reported on twins included in study 11. Studies 2, 7 and 10 (*) did not meet criterion of Systematic Recruitment.

Age range: Studies 1–3: 5–23 years, 26 years, 2–23 years, respectively; Studies 4–6: age range of final sample not specifically reported; Studies 7–12: 4–11 years, 9–12 years, 11–14 years, 6–14 years, 3–14 years and 12–15 years, respectively.

Definition of diagnostic outcomes:

a Autistic Disorder (DSM II R): onset prior to age 5. Criteria: 8 out of 16 items across three categories: at least two difficulties in social interaction category and at least one difficulty in communication and restricted, repetitive and stereotyped patterns of behaviour categories.

b The Broad Phenotype: measured behavioural domains of communication impairment and social dysfunction. Meeting the cut off for either deficit alone or in combination was required for diagnosis. RRB's not included in this criterion.

c Autistic Disorder (DSM IV): onset prior to age 3. Criteria: at least two difficulties in social interaction category and at least one difficulty in communication and restricted, repetitive and stereotyped patterns of behaviour categories. Delays or abnormal functioning in at least one of the following: social interaction, social language communication, and symbolic/imaginative play.

d Atypical Autism: atypical clinical features and a loosened age criterion than used in Autistic Disorder (DSM IV) diagnosis.

e Asperger Syndrome (DSM IV): core triad of symptoms criteria as in Autistic Disorder. However, no clinically significant delay is observed in areas of language development, cognitive development, age‐appropriate self‐skills or adaptive behaviour (other than in social interaction).

f Pervasive Developmental Disorder‐ Not Otherwise Specified (DSM IV): presentations that do not meet the criteria for autistic disorder because of late age of onset, atypical symptomatology, or subthreshold symptomatology, or all of these. This category includes ‘atypical autism’.

g Pervasive Developmental Disorders or Autism Spectrum Disorder (DSM IV): includes conditions of Autistic Disorder, PDD‐NOS, Asperger Disorder, Rett's Disorder, and Childhood Disintegrative Disorder.

h ASD: included individuals with Autistic Disorder diagnosis and those who met a broader definition of ASD based on published criteria for combining information from the ADI‐R and ADOS (see Hallmayer et al., 2011 for more details).

i The Broad Spectrum: classification not based on any validated algorithms but includes individuals that have just missed diagnostic threshold cut offs for ASD diagnosis and exhibit high levels autism traits [see Colvert et al., 2015 for more details].

Ronald and Hoekstra (2011) demonstrated that heritability estimates were high and largely comparable across the published studies. This was true even when the diagnostic criteria for autism were broadened to include ASD – median estimate of proband‐wise concordance for the former was 76% in MZ twins and 0% in dizygotic (DZ), and for the latter 88% and 31%, consistent with a high proportion of heritable effects on ASD. Five further studies have been published since: Hallmayer et al. (2011), Frazier et al. (2014), Nordenbæk, Jørgensen, Kyvik, and Bilenberg (2014), Sandin et al. (2014) and Colvert et al. (2015). However, findings from the recent five studies following the Ronald & Hoekstra review have suggested a more complicated etiological picture.

Both Hallmayer et al. (2011) and Frazier et al. (2014) reported significant influences of shared environmental effects, steering the debate toward the higher importance of the environment rather than a genetic predisposition to ASD. In Hallmayer et al. (2011) the variance of the liability to ASD in a clinical sample was significantly accounted for by shared environmental factors (58%) and only moderately by genetic effects (38%). In Frazier et al. (2014), an ever higher estimate of shared environmental effects was reported: 64–78%, depending on symptom measure. However, they hypothesized that this finding was an effect of the threshold liability model when the assumption of a continuous underlying liability distribution is violated (albeit they were not able to actually test this). In contrast, using a population‐based cohort of ~2 million individuals, Sandin et al. (2014) showed that the individual risk for ASD increased with genetic relatedness, with no effects of shared environment. Two further twin studies (Colvert et al., 2015 and Nordenbæk et al., 2014) again confirmed the importance of genetic effects on ASD by showing high MZ concordance rates of 95% and 94% compared to and 4% & 46% for DZ pairs, respectively, and little support for shared environmental effects.

The potential reasons for the differences across the recent studies are discussed in our latest publication (Colvert et al., 2015) and include issues regarding sample ascertainment and measurement differences. Ronald and Hoekstra (2011) highlighted the fact that diagnoses across studies were often based on unstandardised or proxy measures of ASD rather than the conventional in‐person assessments such as the Autism Diagnostic Observation Schedule (ADOS) (Lord et al., 1989) and Autism Diagnostic Interview – Revised ADI‐R (Lord, Rutter, & Couteur, 1994). Second, since heritability estimates across studies are often derived from selected clinical twin samples prior knowledge of the prevalence (threshold on the liability) is required for statistical modelling. The prevalence for Autism is considered to be 1% in the general population (Baird et al., 2006; Baxter et al., 2015; Brugha et al., 2011; Elsabbagh et al., 2012). However, it is well recognised that the Broad Phenotype cases just falling short of the diagnostic cut off are part of the underlying continuous liability distribution of ASD (Maxwell, Parish‐Morris, Hsin, Bush, & Schultz, 2013). This category is captured by a lower threshold on the liability consistent with a prevalence of around 5%, supported by the fact that 5.8% of general population score above the cut off on the Children Autism Spectrum Test and 1% of these individuals receive an ASD diagnosis (Williams et al., 2005). The use of different thresholds (assumed prevalences) and multiple versus single‐threshold models could be a potential source of descrepancies in estimates of heritability and environmental effects across studies.

By means of a quantile DeFries‐Fulker (DF) extremes analysis on quantitative measures of ASD, Frazier et al. (2014) showed that heritability at the extreme high end of the distribution was more heritable than at the lower parts. This was taken as evidence that the assumption of a multivariate normal distribution (of the genetic component) in ASD was violated and that the liability threshold model was not applicable to this disorder, causing the inflated estimates of C. DF extremes analysis assesses rather than assumes a continuum. If all assumptions of the liability threshold model are correct for a particular disorder, DF extreme heritabilities and those estimated with the liability threshold model will be similar, but only to the extent that the quantitative dimension assessed underlies the qualitative disorder. If they are similar, we can than say that the disorder represents the extreme end of a continuum of ASD symptoms/behaviours rather than being a distinct disorder. If the results are not similar, it means that the disorder under study is different, it does not mean that the assumptions of the liability threshold model that is used to estimate the heritability of qualitative disorders are wrong (Plomin, DeFries, Knopik, & Neiderhiser, 2013).

The aims of this study are fivefold: (a) to reconsider the inconsistent findings, especially with respect to the evidence for shared environmental influences on ASD; (b) to independently estimate twin correlations and heritability estimates for each study while correcting for selection and ascertainment strategy (especially when the original study did not do so); (c) to conduct a meta‐analysis of the published studies using appropriate corrections for selection and ascertainment strategy for each individual study; (d) to study the effects of assumed prevalence rates (fixed thresholds) son twin correlations and heritability estimates in each individual study and on the combined sample; (e) to examine group heritabilities along the distribution of a quantitative ASD measure.

To summarize, in this paper we report results of a quantitative meta‐analysis of the combined data of published twin studies of ASD to date. Many primary twin studies on low prevalence disorders such as ASD are based on ascertained samples of relatively small size. The benefit of the present analysis is not only to produce the best unbiased estimates by applying appropriate ascertainment and selection correction methods but also to increase statistical power to detect effects of small size. In addition to the meta‐analyses, we also explored the etiology along the continuusm of a quantitative ASD measure (Childhood Autism Spectrum Test, Williams et al., 2008) using a population twin sample of 6,413 pairs including ASD affected pairs.

## Methods

### Sample – meta‐analysis

To identify all published studies on heritability of ASD, a PubMed, Science Direct, Google Scholar, and Web of Knowledge computerised search was undertaken to identify any prior reviews of ASD research as well as independent investigations of the topic. This produced all studies reviewed by Ronald and Hoekstra (2011) as well as five studies published after their review, providing a total of 13 eligible studies (Table 1). They were geographically oriented in Northern Europe (UK + Scandinavia), Japan and the United States.

Regarding general inclusion/exclusion criteria, we followed the protocol and guidelines outlined by Sullivan, Kendler, & Neale, 2003 and Cooper, Hedges, & Valentine, 2009). Due to rarity of (clinical) samples of twins with ASD, we aimed to include as many studies as possible. For this reason the criterion for conformity on measurement instruments was loosened, although most included studies employed DSM/ICD diagnostic criteria. To maximise meta‐analysis sample size, data on opposite‐sex twin pairs were also included where reported. When several publications reported on the same sample, we included the most recent report (exclusion criteria for studies 1 and 4 as annotated in Table 1). Study 11 (Sandin et al., 2014) was excluded on the basis of lack of information on the specific number of concordant pairs, and the fact that the majority of twins in this extended family study are most likely reported on in a previous study (Lichtenstein, Carlström, Råstam, Gillberg, & Anckarsäter, 2010), included in the meta‐analyses. Of importance was the Systematic recruitment criterion, which is defined as systematic sampling from a population‐based or hospital register such that affected individuals have an equal probability of being selected. This criterion decisively excluded studies 7 and 10 as they did not systematically select the probands from the general population. In addition, twins in these two studies did not undergo any in‐person screening to validate their diagnosis, a practice followed by every other study included in this meta‐analysis. Study 2 was excluded, since, although using systematic recruitment, it was one biased in favour of families with multiple cases of autism.

Diagnosis blind to zygosity and cotwin's status mostly features in studies conducted after 2000, in line with recent practice in twin research (Sullivan et al., 2003). However, this is not applicable to Random Population Ascertainment (study 8) and in study 9, where proband selection was on the basis of electronic records – an alternative source for systematic recruitment due to technological developments in patient data storage. We retained both of these studies since they met all other criteria.

### Sample – DF extremes analysis

DF extremes analysis was conducted on the Childhood Autism Spectrum Test scores (Williams et al., 2008) collected within the Twins Early Development Study sample when twins were 8 years old. TEDS is a longitudinal study of twins selected from population records of twin births in England and Wales from January 1994 to December 1996. The sample is considered as representative of the population of United Kingdom in terms of maternal ethnicity (92.8%) and educational level (40.1% of mothers has A level qualification or higher) (Haworth, Davis, & Plomin, 2013).

### Statistical analysis

In the present analysis, ASD is treated as a discrete trait and analysed using a liability threshold model. The assumption is that the risk of ASD follows the standard normal distribution with the disorder only manifesting itself when a certain threshold is exceeded. The joint distribution of twin liabilities is assumed to follow the bivariate normal, and the strength between the liabilities is measured by tetrachoric correlations (based on the relative proportions of concordant and discordant pairs). The differences in MZ and DZ correlations provide information on the relative importance of genetic and environmental variance as specified in a standard biometrical genetics model (Neale & Cardon, 1992; Plomin et al., 2013; Rijsdijk & Sham, 2002). The resemblance of MZ and DZ twin pairs is specified as reflecting latent additive genetic factors (A), shared environmental effects (C) and nonshared environmental effects (E). The covariance of MZ pairs is specified as A + C and that of DZ pairs as .5A + C (MZ twins share 100% of segregating genes and DZ twins 50%; and the correlation of 1 for C reflects growing up in the same family). The rationale behind the classical twin design is further outlined in Appendix S1. We assumed a single‐threshold model, with one cut off on the liabilities corresponding to the prevalence of Autism Spectrum Disorder including Asperger syndrome, PDD‐NOS and individuals that score highly on autism symptoms but miss the diagnostic criteria cut off (Colvert et al., 2015) (see Appendix S3). Analyses were conducted in the program Mx (Neale, Boker, Xie, & Maes, 2003).

### Ascertainment correction

Different ascertainment of subjects across the primary studies requires a different correction method (Sullivan et al., 2003). When complete information is available for the sample (i.e. for Random Population Ascertainment, RPA) the normal probability density function is given by:

(1) $$\int_{-\infty }^{+\infty }\int_{-\infty }^{+\infty }\Phi \left(x_{1},x_{2}\right)dx_{1}dx_{2}$$

where Φ is the bivariate normal probability density function of the two liabilities for each twin. The integral signs −∞ to +∞ indicate that the entire distribution is considered. The Mx frequency fit function multiplies the count of each response category by their −2 log likelihood to obtain the overall likelihood of the data.

For nonrandomly selected samples, ascertainment corrections adjust the bivariate normal distribution for the unobserved response categories (therefore, the probability of the observed cells increases), provided that the threshold is known. This is achieved by dividing the RPA samples likelihood function by the probability density of the remaining cells (1 minus the probability density of the missing cells *Ã*). This is equivalent to multiplying the likelihood function by 1/the probability density of the remaining cells: 1/1−*Ã*, which is accomplished by including a weight model.

Under Double (Complete) Ascertainment the correction factor used to multiply the likelihood function (eq (1)) by is:

(2) $$1/1-\int_{-\infty }^{t}\int_{-\infty }^{t}\Phi \left(x_{1},x_{2}\right)dx_{1}dx_{2}$$

where the integral denotes *Ã,* part of the distribution reflecting both twins scoring below threshold for the disorder, i.e. the concordant unaffected pairs.

Under Single Ascertainment the correction factor used to multiply the likelihood function (eq (1)) by is:

(3) $$1/1-\int_{-\infty }^{t}\Phi \left(x_{1}\right)dx_{1}$$

where the integral denotes *Ã*, part of the distribution reflecting the first twin (proband) is below threshold for the disorder (unaffected), i.e. individual which come to the attention of the study (probands) must be affected.

The correction for Incomplete Ascertainment (mix of ‘singly’ (S) and ‘doubly’ (D) ascertained concordant pairs) is dependent on *π*. Then, the ascertainment probability is the proportion of formally diagnosed probands who were originally identified as ‘at risk’, or 2D/2D + S^15^. In practice (following Sullivan et al., 2003), the likelihood of the discordant pairs as well as the singly ascertained concordant pairs is corrected with the following weight function, incorporating *π*:

(4) $$\pi /2\pi \int_{t}^{+\infty }\Phi \left(x_{1}\right)dx_{1}-\pi^{2}\int_{t}^{+\infty }\int_{t}^{+\infty }\Phi \left(x_{1},x_{2}\right)dx_{1}dx_{2}$$

The likelihood of the doubly ascertained concordant pairs is corrected by weight:

(5) $$2\pi -\pi^{2}/2\pi \int_{t}^{+\infty }\Phi \left(x_{1}\right)dx_{1}-\pi^{2}\int_{t}^{+\infty }\int_{t}^{+\infty }\Phi \left(x_{1},x_{2}\right)dx_{1}dx_{2}$$

Estimates of *π* for IA are either provided by the studies or computed by utilising information available in the publications.

Data files were generated for each of the primary studies in Table 1, including the frequencies of each available response category, *π* and the threshold *z*‐values corresponding to the reported prevalences in the individual studies. When not provided, we used a prevalence of 5% [fixed *z*‐value of 1.65] for ASD (Baird et al., 2006). The data (Data S1) are available to view online. Individual study analyses were followed by meta‐analytic analyses, by fitting one overall MZ and DZ correlation or one overall set of A, C and E parameters to the data, while applying appropriate weight corrections and using fixed thresholds for each study if needed. An example script (Appendix S2) is available as online supplementary material.

### Quantile DF extremes analysis

In contrast to variance decomposition analysis used in the classical twin study, DF extremes analysis (DeFries & Fulker, 1985) is a method based on a simple multiple regression in which cotwin's score on a quantitative dimension is predicted from the proband score and the pair's coefficient of genetic relatedness. The proband can be either an affected individual or an extreme scorer on the dimension. Differential regression for the MZ and DZ cotwins toward the mean of the unselected population is then used as a test of genetic influence. These estimates are referred to as ‘group heritabilities’. Different cuts (represented by z‐scores) along the distribution of the quantitative dimension can be made to identify probands, allowing examination of etiologic influences along the entire continuum (quantile DF regression). We used the reframed model‐fitting version of the basic model (Purcell & Sham, 2003) applied to a population twin data set of Childhood Autism Spectrum Test scores measured at age 8. The sample consisted of 2256 MZ and 4157 DZ pairs, including affected twins from the Social Relationship Study (see Colvert et al., 2015 for more details on the sample). We estimated group heritabilities by applying on transformed scores different cuts ranging from z‐values −1.28 to 2.33 (corresponding to a cumulative probability at the right‐hand side of 90–1%).The model‐fitting framework enables the generation of maximum likelihood 95% Confidence Intervals around the group heritabilities, rather than using adjusted standard errors. We (Trzaskowski & Rijsdijk) have recently reprogrammed the Purcell scripts in R and OpenMx (available upon request or downloadable from the OpenMx website, http://openmx.psyc.virginia.edu/thread/2384).

## Results

### Tetrachoric correlations

**Figure 1:**
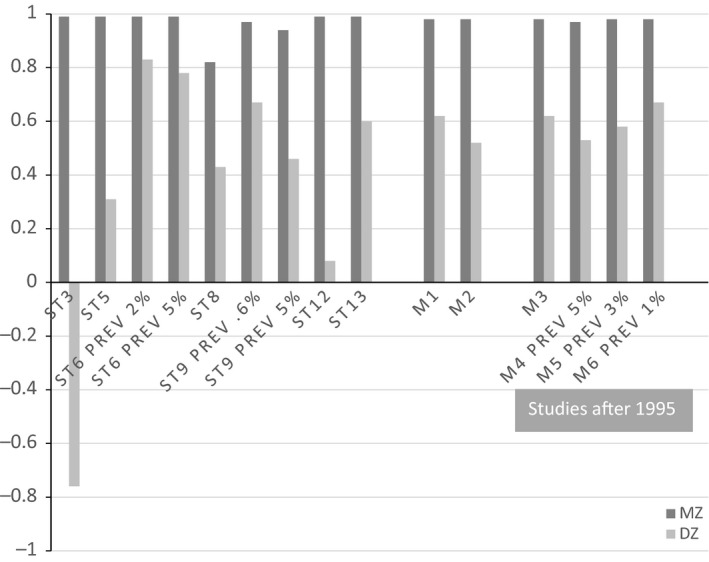
Maximum likelihood MZ and DZ tetrachoric correlation coefficients for each of the studies individually as well as meta‐analysis results using 6 different configurations (M1–M6). Meta‐analysis 1: using all data and reported prevalence as fixed thresholds. Meta‐analysis 2: as in 1 but changing prevalence of Autism Spectrum Disorder to 5% in Study 6 & Study 9. In Meta‐analysis 3–6 only studies after 1995 using the Broad Phenotype definitions were considered. Meta‐analysis 3: using reported prevalence as fixed thresholds, Meta‐analysis 4: fixing all thresholds to 5%; Meta‐analysis 5: fixing all thresholds to 3% and Meta‐analysis 6: fixing all thresholds to 1%. Note that in all analyses, the threshold of study 8 (Random Population Ascertained sample) was estimated (z‐value around 2.4 corresponding to a 0.08% prevalence)

Point estimates for MZ and DZ tetrachoric correlations for individual studies as well as meta‐analytic results (corrected for incomplete ascertainment and selection) are shown in Figure 1 and presented with 95% Confidence Intervals [95% CI] in Table S1. To deal with uncertainties concerning the definitions used to select the ASD phenotype and corresponding prevalence to fix the thresholds, we conducted several meta‐analyses.

The first was on all selected studies using fixed thresholds based on prevalences as reported in each study (see Data S1), aside from study 8 for which the threshold is always estimated: *r*_MZ_ = 0.98 (95% CI 0.97–0.99), *r*_DZ_ = 0.62 (95% CI 0.55–0.68). In the second meta‐analysis, the thresholds of study 6 and 9 were fixed to a 5% prevalence (*z*‐value 1.65) which is more in line with the Broad Phenotype definition: *r*_MZ_ = 0.98 (95% CI 0.96–0.99), *r*_DZ_ = 0.52 (95% CI 0.44–0.60). Next, only studies conducted after 1995, signifying the awareness of the Broad Phenotype definition, were included. In that meta‐analysis, we fixed thresholds as reported in each study but estimated the threshold for study 8: *r*_MZ_ = 0.98 (95% CI 0.96–0.99), *r*_DZ_ = 0.62 (95% CI 0.55–0.68). Finally, again considering the studies conducted after 1995, we fixed all prevalences to 5%, 3% and 1%, respectively, to test the range of values reported for ASD and the Broad Phenotype in the literature. In effect, we found that as the prevalence rate decreased from 5% to 3% to 1%, the DZ correlations increased.

### A, C and E estimates

**Figure 2:**
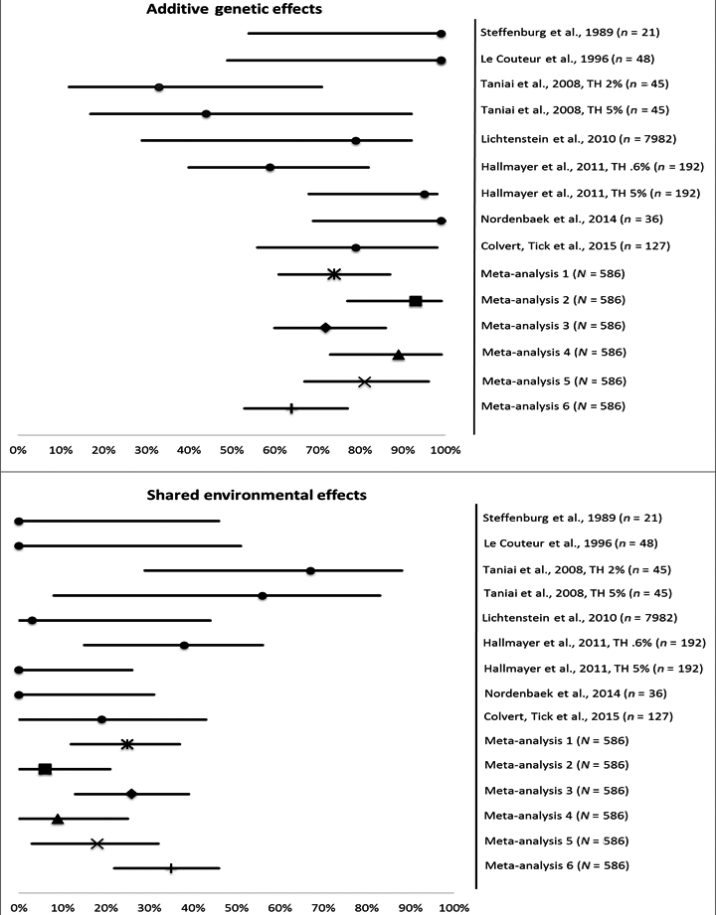
Forest plots, upper panel = additive genetic effects (A), lower panel = shared environmental effects (C), calculated for each study individually as well meta‐analysis estimates, using 6 different configurations. Horizontal lines represent the 95% confidence intervals. Meta‐analysis 1: using all data and reported prevalence as fixed thresholds. Meta‐analysis 2: as in 1 but changing prevalence of Autism Spectrum Disorder to 5% in Study 6 & Study 9. In Meta‐analysis 3–6 only studies conducted after 1995 using broader phenotype definitions were considered. Meta‐analysis 3: using reported prevalence as fixed thresholds, Meta‐analysis 4: fixing all thresholds to 5%; Meta‐analysis 5: fixing all thresholds to 3% and Meta‐analysis 6: fixing all thresholds to 1%. Note that in all analyses, the threshold of study 8 (Random Population Ascertained sample) was estimated (z‐value around 2.4 corresponding to a 0.08% prevalence)

The A, C and E point estimates (corrected for incomplete ascertainment and selection) can be found in Table S2. The forest plot (Figure 2), depicts in the first panel the additive genetic effects (A) and in the second the shared environmental effects (C) for individual studies as well as for the meta‐analyses. Estimates for additive genetic effects were generally high with exception of two studies that showed significant proportions of C (studies 6 and 9). However, when the threshold for study 9 was changed to a prevalence rate of 5%, the C estimate dropped to zero.

The first meta‐analysis on all selected studies, using fixed thresholds as reported in each study, yielded a heritability of 74% (95% CI 0.70–0.87), with a significant proportion of shared environmental effects: 25% (95% CI 0.12–0.37). In the second meta‐analysis, when the thresholds of study 6 and 9 were fixed to 5% prevalence, the heritability increases to 93% (95% CI 0.77–0.99) and C becomes nonsignificant. A detailed investigation of studies conducted after 1995, in which the Broad Phenotype definition for ASD was included, applying thresholds as reported in each study gave estimates similar to when all studies were considered. However, when we subsequently fixed all thresholds to either 5%, 3% or 1%, we saw an increase in the proportion of C (consistent with the observed increase in DZ correlations relative to the MZ correlations, Figure 1). This is a significant finding that stresses the importance of the assumed prevalences of the disorder in the population when using model‐fitting analysis on ascertained samples. Note that in all analyses the threshold for study 8 (RPA sample) was estimated.

### Excluding DZ opposite‐sex pairs

To investigate how inclusion of the DZ opposite‐sex pairs might have influenced the overall results, Models 4, 5 and 6 in Figure 1 were repeated excluding these pairs. As expected, the MZ correlations did not change. The DZ correlations (point estimates) increased, but not significantly so for the analyses using 5% and 3% prevalence: .69 (.60/.77) compared to .53 (.44/.66) and .74 (.65/.81) compared to .58 (.51/.65) due to overlapping 95% CI.

For the analyses using 1% prevalence, the 95% CI were nonoverlapping: .79 (.73/.85) compared to .67 (.61/.72), meaning that C was significantly different (61% vs. 35%). Overall, however, these results did not change the conclusion that the DZ correlation (and the power to detect C) increases as a function of increasing the fixed threshold in the liability model.

### DeFries‐Fulker extreme analyses

**Figure 3:**
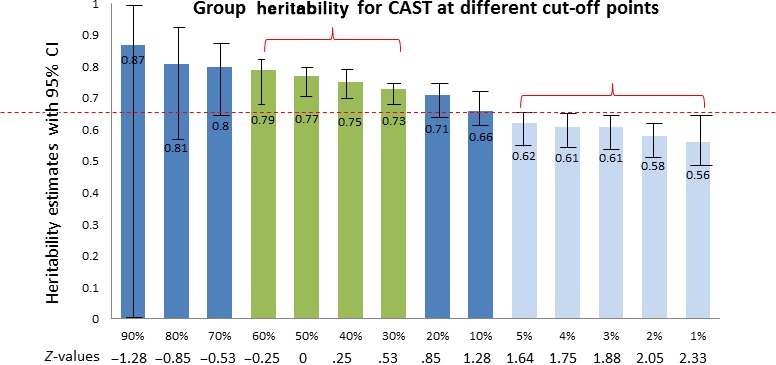
DF group heritabilities of the Childhood Autism Spectrum Test scores at age 8. The *x*‐axis denotes the cut off points applied to generate the groups for the DF analyses: the proportions represent the cumulative probability at the right‐hand side of the distribution, using the z‐values below as cut off points. The horizontal line drawn at and above the upper 95% CI of the group heritabilities at the higher end of the distribution (5–1%), serves as a visual guide to illustrate the groups which the high end estimates are not overlapping with

Figure 3 shows the results of the group heritabilities estimated along the continuum of the CAST scores measured in 12,826 individuals. For the most part, we see no strong departure from linearity (differences in heritabilities across the distribution), but in so far as there is a departure it occurs in the middle and not at the ends of the spectrum (nonoverlapping 95% CI show that there were no significant differences in heritability between the high and lower extremes). In addition, the effects are the opposite of what has been previously assumed (i.e. lower point estimates of group heritabilities at the higher end), and consequently requires further study.

## Discussion

Using a quantitative meta‐analytic approach, we estimated the heritability of ASD using studies of twins with a (clinical) diagnosis of autism spectrum disorder. Applying appropriate ascertainment corrections and maximum likelihood estimation, our study produced tetrachoric twin correlations and heritability estimates for all studies published to date, inclusive of studies that previously only reported proband‐wise concordant rates. The meta‐analytic heritability estimates ranged between 64% and 91% (despite diagnostic heterogeneity) and are in line with previous reports (Bailey et al., 1995; Colvert et al., 2015; Folstein & Rutter, 1977; Le Couteur et al., 1996; Lichtenstein et al., 2010; Nordenbæk et al., 2014; Ritvo, Freeman, Mason‐Brothers, Mo, & Ritvo, 1985; Rosenberg et al., 2009; Steffenburg et al., 1989; Taniai, Nishiyama, Miyachi, Imaeda, & Sumi, 2008).

The most important statistical finding concerns the assumptions we make about the underlying distribution of the phenotype as a discrete trait, which is a standard normal distribution with a threshold discriminating between affected and nonaffected individuals. A statistical correction necessary for selected (clinical) samples is fixing the threshold in the model to the prevalence rate of the disorder in the general population. The different diagnostic outcomes across ASD studies prove problematic to derive the correct overall prevalence in meta‐analysis. When looking at the individual studies as well as the meta‐analysis results, it is apparent that detecting significant heritable variance is quite robust, but detecting significant C effects depends on the assumed prevalence of the disorder. Effectively, pushing the threshold to higher values (i.e. decreasing the assumed prevalence rate) will not affect the MZ twin correlations as much since they are already at the top of their statistical bound (upper 95% CI values approaching 1). However, the DZ correlations will increase relative to the MZ correlations, consistent with increasing the effect of shared environment. Given the importance of this effect, fitting multiple‐threshold models including a Broader Phenotype as a meaningful subcategory with a lower (fixed) threshold on the spectrum to ASD (Colvert et al., 2015; Sasson, Lam, Parlier, Daniels, & Piven, 2013) might perhaps be a better method, albeit there is no generally agreed measure of such a category.

A second important point to note from the meta‐analyses is that even when shared environmental effects become significant, they never explain the majority of the variance in ASD (as claimed by Hallmayer et al., 2011 and Frazier et al., 2014). We therefore conclude that significance of shared environments (C) in ASD is likely to be a statistical artefact as a result of the assumptions made of the prevalence in addition to oversampling of DZ concordant pairs. The meta‐analysis results are in line with the results from the largest extended family population study (Sandin et al., 2014), (showing no effects of shared environment) as well as results from the only random population twin study, which did not need to rely on fixed threshold correction (Lichtenstein et al., 2010). Nevertheless, we note here the limitation of random population studies, which use proband selection based on electronic records within registries rather than using cases with individually confirmed clinical diagnosis of ASD.

A third point we would like to make is that there is not much evidence for nonlinearity of heritability across the distribution of a quantitative ASD measure, which suggests that ASD as disorder can be conceived as the extreme of ASD symptoms/behaviours rather than being a distinct disorder (albeit replication in bigger samples including more affected pairs and using different ASD measures would be preferable). The only way to determine whether ASD as a disorder fits the characteristics of a polygenetic trait is via molecular genetic studies. There is evidence for the contribution of both common variants and rare mutations (Gaugler et al., 2014; Iossifov et al., 2015) and at this point it is simply not possible to definitively decide on one model over the other. If we take the totality of evidence in to account, common polymorphic variants are important in determining genetic variance in ASD. The liability threshold model used in twin analyses cannot be rejected based on current evidence and unlikely to be a plausible alternative for the observed fluctuations in shared environment across studies.

## Conclusion

Using an appropriate meta‐analytic statistical approach we demonstrated that the etiology of ASD in a combined sample is more consistent with strong genetic influences. Second, we can reject the claim that there is a strong shared environmental effect on autism spectrum disorders accounting for the majority of variance and alert to the danger of placing too much weight on findings from a single study, such as Hallmayer et al. (2011).

At the same time, we *do not* exclude the possibility that environmental, or at least nongenetic, effects influence ASD. But unless a suitably powered and well‐designed new study comes forward, this claim should be put to one side for now.

**Key points**

- Two recent studies point toward importance of shared environments in ASD.
- This effect is potentially a statistical artefact due to overinclusion of concordant DZ twins.
- Differential prevalence assumptions can alter heritability estimates.
- Clinically recognised Broad Phenotype ought to be recognised in statistical modelling by fitting multiple thresholds to reflect the quantitative genetic risk for ASD.

## Supporting information


**Appendix S1.** The classical twin method.

**Appendix S2.** Example of Meta‐Analysis Mx script.

**Appendix S3.** Meta‐Analysis Data.

**Table S1.** Maximum likelihood estimates of the MZ and DZ twin correlations.

**Table S2.** Maximum likelihood estimates of the genetic and environmental variance components.
