# Supplementary material for: Heritability of autism spectrum disorders: a meta‐analysis of twin studies
Source: J Child Psychol Psychiatry. 2015 Dec 27;57(5):585–95. doi: 10.1111/jcpp.12499 (PMC4996332; doi:10.1111/jcpp.12499)
Supplement: Supplementary file 1 — Appendix S1. The classical twin method. [file JCPP-57-585-s001.docx]

**The Classical Twin Method**

Behavioural Genetics examines the genetic basis of behavioural phenotypes (disorders and dimensional traits) using differences in genetic correlation between relative pairs. The classical twin method is the most popular design in behavioural genetics. The existence of two types of twin pairs, monozygotic (MZ) and dizygotic (DZ) reared together provides a natural experiment for untangling genetic from environmental effects. Since MZ twins are developed from the same fertilised ovum they are genetically identical, whereas DZ twins developed from two separate fertilised ova share on average 50% of their segregating genes. The observed covariances of these pairs can be expressed in a mathematical model in terms of correlated latent genetic and environmental factors. The latent factors that are considered are:

• Additive genetic influences, A, represent the sum of the effects of the individual alleles at all loci that influence the trait.

• Non-additive genetic influences, D, which represent interactions between alleles of the same marker (dominance genetic variation) or different markers (epistasis).

• Environmental influences, C, that make family members more alike (common environmental variation), e.g. socio-economic status, parenting style, diet.

• Unique environmental influences, E, that result in differences among members of one family e.g. accidents, differential parental treatment, as well as measurement error.

According to biometrical genetics, the MZ and DZ twins have different degrees of correlation for the genetic components A (1 vs .50) and D (1 vs .25) but the same degree of correlation for the environmental components C (1 in both MZ and DZ pairs) and E (0 in both MZ and DZ pairs). Since D involves the interaction effects between alleles, for this source of variance to explain the correlation between relatives, they would need to have the same alleles identical by decent (IBD, alleles from same parental origin), which is 100% the case for MZ pairs, only 25% for siblings/DZ twins and 0% for any other relative pair. Assuming that MZ and DZ twins experience the same degree of similarity in their environments, then any excess of similarity between MZ (compared to DZ) twins can be interpreted as due to the greater proportion of genes shared by MZ twins, and thus, gives us an estimate for A. An estimate for C is given by the difference in MZ correlation and the estimated effect of A. Differences in MZ twins can only be due to unique environmental influences and, thus, gives us an estimate for E. Note that C and D are confounded in the classical twin study and cannot be estimated simultaneously. The twin correlations indicate which of the two components is more likely to be present. When DZ correlations are less than half the MZ correlations, dominance is indicated, because D correlates perfectly for MZ but only 25% for DZ twin pairs. Common environmental influences, on the other hand, will make the DZ correlations greater than half the MZ correlations. Therefore, DZ correlations about half the MZ correlations suggest additive genetic influences, but are not inconsistent with the presence of both C and D. In other words, data on twins reared together do not contain enough information to tease out the contrasting effects of C and D. If data of adoptive siblings are included (giving us an independent estimate of C) we can estimate both sources of variance. The estimates are solved using covariance structure analysis with maximum likelihood estimation in Structural Equation Modeling software (Neale & Cardon, 1992).

*Assumptions:* There are a number of assumptions made in the classical twin method. It is important to be aware of the implications of these and consider whether they are realistic in relation to the trait under study. The assumptions are that: (i) MZ and DZ twin pairs share their

environments to the same extent; (ii) Gene-environment correlations (passive/active) and interactions are minimal for the trait in question (if not they get incorporated into the other variance components, e.g. GxE interaction effects will increase the E variance, but GxC interaction will increase the heritability estimate) (iii) Twins are no different from the general population. Procedures for testing these assumptions are discussed in detail elsewhere (Rijsdijk & Sham, 2002; Neale & Cardon, 1992). When the assumptions are met, the classical twin design is the most powerful tool for investigating the relative contribution of genetic and environmental influences to individual differences in traits.

**The Liability Threshold Model**

The behavioural phenotypes the method is concerned with are traits that are the result of multiple genes and environmental factors of small effect size, as explained by a polygenic model (Plomin et al., 2001; Neale & Cardon, 1992). The cumulative effects of multiple genes lead to a continuous (normal) distribution, which fits well with quantitative characteristics, but is not directly applicable to categorical traits. The liability-threshold model proposes that many genes of small effect exert their influence on an unobserved normally distributed variable - known as a liability - and that the disease develops if this liability exceeds a certain threshold value. Variance components models as described above can then be applied to categorical twin data by assuming that the ordered categories reflect an imprecise measurement of an underlying normal distribution of liability with one or more thresholds (cut-offs) to discriminate between the ordered classes. The joint distribution of two categorical traits (twin 1 and twin 2

variables) is assumed to follow a bivariate normal distribution, the shape determined by the relative proportions of twin pairs concordant and discordant for the observed categories (estimating tetrachoric correlations). The relative difference in derived MZ and DZ tetrachoric correlations will further inform on the usual twin method of variance decomposition, leading to an estimate of the heritability of the (liability to the) disorder. Under the ACE model, each of the three components contributing to the liability is assumed to follow a normal distribution. Deviation from the assumed normal distribution will violate the bivariate normal assumption, the extent to which depending on the severity of the deviation (Benchek & Morris, 2014).

Neale MC & Cardon LR (1992). Methodology for Genetic Studies of Twins and Families, Dordrecht: Kluwer Academic Publishers.

Plomin R., DeFries JC, McClearn GE & McGuffin P (2001). Behavioral Genetics. 4th Edition, New York: Worth Publishers.

Rijsdijk FV & Sham PC (2002). Analytic approaches to twin data using structural equation models. Briefings in Bioinformatics, 3, 119–133.

Benchek PH & Morris NJ (2013). How meaningful are heritability estimates of liability? Hum Genet. 132(12), 1351-1360.
