# Supplementary material for: Heritability of autism spectrum disorders: a meta‐analysis of twin studies
Source: J Child Psychol Psychiatry. 2015 Dec 27;57(5):585–95. doi: 10.1111/jcpp.12499 (PMC4996332; doi:10.1111/jcpp.12499)
Supplement: Supplementary file 2 — Appendix S2. Example of Meta‐Analysis Mx script. [file JCPP-57-585-s002.docx]

! Mx Script to estimate meta-analysis ACE components for ASD clinical diagnosis

#ngroups 16

G1: Model parameters

Calc

Begin Matrices;

X full 1 1 FREE ! A path

Y full 1 1 FREE ! c path

Z full 1 1 FREE ! E path

H full 1 1

U unit 1 1 ! flag 1

O zero 1 1 ! flag 0

P full 1 1 ! definition var 'ascertainment probability' (pi-hat) of each study

T full 1 1 ! definition var 'threshold' of each study

End Matrices;

Matrix H .5

Start .8 X 1 1

Start .5 Y 1 1

Start .5 Z 1 1

Begin Algebra;

A= X*X';

C= Y*Y';

E= Z*Z';

S= (P) _ (P) _ P+P-P*P ; ! Vector of ascertainment terms

L= A+C+E | A+C _ A+C | A+C+E ; ! Exp MZ cov matrix

M= A+C+E | H@A+C _ H@A+C | A+C+E ; ! Exp DZ cov matrix

J= (P+P)*\mnor(U_O_T_T_U)-P*P*\mnor(L_O|O_T|T_T|T_U|U);

K= (P+P)*\mnor(U_O_T_T_U)-P*P*\mnor(M_O|O_T|T_T|T_U|U);

End Algebra:

interval A 1 1 C 1 1 E 1 1

Options RSidual

End

! *****************************************************

! Study 3 Steffenburg 1989 CA (pi=1) TH=2.55

! *****************************************************

G2: MZ twin pairs

Data Ninput=9

Labels studyid studyyr zygosity twin1 twin2 asc freq pihat threshold

Ordinal File=St3.dat

Select if zygosity = 1;

Select twin1 twin2 asc freq pihat threshold;

Definition Asc Freq Pihat Threshold;

Begin Matrices= Group 1;

F full 1 1 ! definition var 'Frequency' for each pair type

G full 1 4 ! definition var to select appropriate ascertainment term in mx S

End Matrices;

Specify P Pihat

Specify T Threshold

Specify F Freq

Matrix G 1 1 1 1

Specify G Asc 0 Asc 0

Covariances L;

Thresholds T|T;

Frequency F;

Weight \part(S,G)%J;

Options RSidual

End

G3: DZ twin pairs

Data Ninput=9

Labels studyid studyyr zygosity twin1 twin2 asc freq pihat threshold

Ordinal File=St3.dat

Select if zygosity = 2;

Select twin1 twin2 asc freq pihat threshold;

Definition Asc Freq Pihat Threshold;

Begin Matrices= Group 1;

F full 1 1

G full 1 4

End Matrices;

Specify P Pihat

Specify T Threshold

Specify F Freq

Matrix G 1 1 1 1

Specify G Asc 0 Asc 0

Covariances M;

Thresholds T|T;

Frequency F;

Weight \part(S,G)%K;

Options RSidual

End

! ********************************************

! Study 5 Le Couteur 1996 CA (pi=1) TH=1.65

! ********************************************

G4: MZ twin pairs

Data Ninput=9

Labels studyid studyyr zygosity twin1 twin2 asc freq pihat threshold

Ordinal File=St5.dat

Select if zygosity = 1;

Select twin1 twin2 asc freq pihat threshold;

Definition Asc Freq Pihat Threshold;

Begin Matrices= Group 1;

F full 1 1

G full 1 4

End Matrices;

Specify P Pihat

Specify T Threshold

Specify F Freq

Matrix G 1 1 1 1

Specify G Asc 0 Asc 0

Covariances L;

Thresholds T|T;

Frequency F;

Weight \part(S,G)%J;

Options RSidual

End

G5: DZ twin pairs

Data Ninput=9

Labels studyid studyyr zygosity twin1 twin2 asc freq pihat threshold

Ordinal File=St5.dat

Select if zygosity = 2;

Select twin1 twin2 asc freq pihat threshold ;

Definition Asc Freq Pihat Threshold ;

Begin Matrices= Group 1 ;

F full 1 1

G full 1 4

End Matrices;

Specify P Pihat

Specify T Threshold

Specify F Freq

Matrix G 1 1 1 1

Specify G Asc 0 Asc 0

Covariances M;

Thresholds T|T;

Frequency F;

Weight \part(S,G)%K ;

Options RSidual

End

! **********************************************

! Study 6 Taniai 2008 CA (pi=1) TH=2.06

! **********************************************

G6: MZ twin pairs

Data Ninput=9

Labels studyid studyyr zygosity twin1 twin2 asc freq pihat threshold

Ordinal File=St6.dat

Select if zygosity = 1;

Select twin1 twin2 asc freq pihat threshold;

Definition Asc Freq Pihat Threshold;

Begin Matrices= Group 1;

F full 1 1

G full 1 4

End Matrices;

Specify P Pihat

Specify T Threshold

Specify F Freq

Matrix G 1 1 1 1

Specify G Asc 0 Asc 0

Covariances L;

Thresholds T|T;

Frequency F;

Weight \part(S,G)%J;

Options RSidual

End

G7: DZ twin pairs

Data Ninput=9

Labels studyid studyyr zygosity twin1 twin2 asc freq pihat threshold

Ordinal File=St6.dat

Select if zygosity = 2;

Select twin1 twin2 asc freq pihat threshold;

Definition Asc Freq Pihat Threshold;

Begin Matrices= Group 1;

F full 1 1

G full 1 4

End Matrices;

Specify P Pihat

Specify T Threshold

Specify F Freq

Matrix G 1 1 1 1

Specify G Asc 0 Asc 0

Covariances M;

Thresholds T|T;

Frequency F;

Weight \part(S,G)%K;

Options RSidual

End

! *******************************************************************

! Study 8 Lichtenstein 2010 RAP, no corrections AT ALL, TH estimated

! *******************************************************************

G8: MZ twin pairs

Data Ninput=9

Labels studyid studyyr zygosity twin1 twin2 asc freq pihat threshold

Ordinal File=St8.dat

Select if zygosity = 1;

Select twin1 twin2 freq;

Definition Freq ;

Begin Matrices= Group 1;

V full 1 1 FREE ! Threshold

F full 1 1

End Matrices;

Specify F Freq

Covariances L;

Thresholds V|V ;

Frequency F ;

MA V 2.3 ! start value for Threshold

Options RSidual

End

G9: DZ twin pairs

Data Ninput=9

Labels studyid studyyr zygosity twin1 twin2 asc freq pihat threshold

Ordinal File=St8.dat

Select if zygosity = 2;

Select twin1 twin2 freq ;

Definition Freq ;

Begin Matrices= Group 1;

V full 1 1 =V8

F full 1 1

End Matrices;

Specify F Freq

Covariances M;

Thresholds V|V ;

Frequency F ;

Options RSidual

End

! *********************************************************

! Study 9 Hallmayer 2011 IA (pi=.92) TH=2.49

! *********************************************************

G10: MZ twin pairs

Data Ninput=10

Labels rep studyid studyyr zygosity twin1 twin2 asc freq pihat threshold

Ordinal File=St9.dat

Select if zygosity = 1;

Select twin1 twin2 asc freq pihat threshold;

Definition Asc Freq Pihat Threshold;

Begin Matrices= Group 1;

F full 1 1

G full 1 4

End Matrices;

Specify P Pihat

Specify T Threshold

Specify F Freq

Matrix G 1 1 1 1

Specify G Asc 0 Asc 0

Covariances L;

Thresholds T|T;

Frequency F;

Weight \part(S,G)%J;

Options RSidual

End

G11: DZ twin pairs

Data Ninput=9

Labels studyid studyyr zygosity twin1 twin2 asc freq pihat threshold

Ordinal File=St9.dat

Select if zygosity = 2;

Select twin1 twin2 asc freq pihat threshold;

Definition Asc Freq Pihat Threshold;

Begin Matrices= Group 1;

F full 1 1

G full 1 4

End Matrices;

Specify P Pihat

Specify T Threshold

Specify F Freq

Matrix G 1 1 1 1

Specify G Asc 0 Asc 0

Covariances M;

Thresholds T|T;

Frequency F;

Weight \part(S,G)%K;

Options RSidual

End

! *********************************************

! Study 12 Nordenbaek 2014 IA (pi=.76) TH=2.49

! *********************************************

G12: MZ twin pairs

Data Ninput=9

Labels studyid studyyr zygosity twin1 twin2 asc freq pihat threshold

Ordinal File=St11.dat

Select if zygosity = 1;

Select twin1 twin2 asc freq pihat threshold;

Definition Asc Freq Pihat Threshold;

Begin Matrices= Group 1;

F full 1 1

G full 1 4

End Matrices;

Specify P Pihat

Specify T Threshold

Specify F Freq

Matrix G 1 1 1 1

Specify G Asc 0 Asc 0

Covariances L;

Thresholds T|T;

Frequency F;

Weight \part(S,G)%J;

Options RSidual

End

G13: DZ twin pairs

Data Ninput=9

Labels studyid studyyr zygosity twin1 twin2 asc freq pihat threshold

Ordinal File=St11.dat

Select if zygosity = 2;

Select twin1 twin2 asc freq pihat threshold;

Definition Asc Freq Pihat Threshold;

Begin Matrices= Group 1;

F full 1 1

G full 1 4

End Matrices;

Specify P Pihat

Specify T Threshold

Specify F Freq

Matrix G 1 1 1 1

Specify G Asc 0 Asc 0

Covariances M;

Thresholds T|T;

Frequency F;

Weight \part(S,G)%K;

Options RSidual

End

! ****************************************************************************

! Study 13 Colvert & Tick 2014 PA no corrections but TH fixed to 1.65 (5% Prev)

! ****************************************************************************

G14: MZ twin pairs

Data Ninput=9

Labels studyid studyyr zygosity twin1 twin2 asc freq pihat threshold

Ordinal File=St12.dat

Select if zygosity = 1;

Select twin1 twin2 asc freq pihat threshold;

Definition Asc Freq Pihat Threshold;

Begin Matrices= Group 1;

F full 1 1

G full 1 4

End Matrices;

Specify P Pihat

Specify T Threshold

Specify F Freq

Matrix G 1 1 1 1

Specify G Asc 0 Asc 0

Covariances L;

Thresholds T|T ;

Frequency F ;

Options RSidual

End

G15: DZ twin pairs

Data Ninput=9

Labels studyid studyyr zygosity twin1 twin2 asc freq pihat threshold

Ordinal File=St12.dat

Select if zygosity = 2;

Select twin1 twin2 asc freq pihat threshold ;

Definition Asc Freq Pihat Threshold ;

Begin Matrices= Group 1 ;

F full 1 1

G full 1 4

End Matrices;

Specify P Pihat

Specify T Threshold

Specify F Freq

Matrix G 1 1 1 1

Specify G Asc 0 Asc 0

Covariances M;

Thresholds T|T ;

Frequency F ;

Options RSidual nd=4

End

G16: constrain Total variance A+C+E to 1

Constraint NI=1

Begin Matrices = Group 1;

End Matrices;

Constraint U = A+C+E;

End
