# Supplementary material for: Heritability of autism spectrum disorders: a meta‐analysis of twin studies
Source: J Child Psychol Psychiatry. 2015 Dec 27;57(5):585–95. doi: 10.1111/jcpp.12499 (PMC4996332; doi:10.1111/jcpp.12499)
Supplement: Supplementary file 4 — Table S1. Maximum likelihood estimates of the MZ and DZ twin correlations. [file JCPP-57-585-s004.docx]

**Table S1** Maximum likelihood estimates of the MZ and DZ twin correlations (with 95% CI) for each of the individual studies as well as Meta-analysis estimates based on 6 different configurations.

| Studies | Reference | R_MZ_ | R_DZ_ |
| --- | --- | --- | --- |
| Study 3 | Steffenburg et al., 1989 | .99 (.98 / 1.00) | -.76 (-.98 / .77) |
| Study 5 | Le Couteur et al., 1996 | .99 (.99 / 1.00) | .31 (-.09 / .75) |
| Study 6_1 | Taniai et al., 2008, prev 2%^^^ | approaches 1 | approaches .83 |
| Study 6_2 | Taniai et al., 2008, prev 5%^^^ | approaches 1 | approaches .78 |
| Study 8 | Lichtenstein et al., 2010* | .82 (.65 / .92) | .43 (.20 / .61) |
| Study 9_1 | Hallmayer et al., 2011, prev 0.6% | .97 (.94 / .99) | .67 (.56 / .77) |
| Study 9_2 | Hallmayer et al., 2011, prev 5% | .94 (.89 / .98) | .46 (.30 / .60) |
| Study 12 | Nordenbaek et al., 2014, prev 5% | .99 (.97 / 1.00) | .08 (-.39 / .53) |
| Study 13 | Colvert, Tick et al., 2015 | .99 (.95 / 1.00) | .60 (.46 / .71) |
|  |  |  |  |
| Study  3, 5, 8, 12, 13,  6_1, & 9_1 | Meta-analysis, using reported prevalence as fixed TH (TH in St8 estimated) | .98 (.97 / .99) | .62 (.55 / .68) |
| Study  3, 5, 8, 11, 12,  6_2, & 9_2 | Meta-analysis, changing prevalence of ASD to 5% in St6 & St9 (TH in St8 estimated) | .98 (.96 / .99) | .52 (.44 / .60) |
|  |  |  |  |
| Study  5, 8, 12, 13  6_1 & 9_1 | Meta-analysis, studies after 1995 using broader phenotype, using reported prevalence as fixed TH (TH in St8 estimated) | .98 (.96 / .99) | .62 (.55 / .68) |
| Study  5, 6, 8, 9, 12, 13 | Meta-analysis, studies after 1995 using broader phenotype, using prevalence of **5% for all** (TH st8 estimated) | .97 (.96 / .99) | .53 (.44 / .60) |
| Study  5, 6, 8, 9, 12, 13 | Meta-analysis, studies after 1995 using broader phenotype, using prevalence of 3**% for all** (TH St8 estimated) | .98 (.96 / .99) | .58 (.51 / .65) |
| Study  5, 6, 8, 9, 12, 13 | Meta-analysis, studies after 1995 using broader phenotype, using prevalence of **1% for all** (TH St8 estimated) | .98 (.97 / .99) | .67 (.61 / .72) |

* Threshold for Study 8 estimated at around 2.40 z-score, equivalent to prevalence value of 0.08%.

^^^ Due to non-convergence of the correlation model (MZ correlations approaching 1) the tetrachoric correlations are derived from the ACE estimates.
