# Supplementary material for: Heritability of autism spectrum disorders: a meta‐analysis of twin studies
Source: J Child Psychol Psychiatry. 2015 Dec 27;57(5):585–95. doi: 10.1111/jcpp.12499 (PMC4996332; doi:10.1111/jcpp.12499)
Supplement: Supplementary file 5 — Table S2. Maximum likelihood estimates of the genetic and environmental variance components. [file JCPP-57-585-s005.docx]

**Table S2** Maximum likelihood estimates of the genetic and environmental variance component (with 95% CI) for each of the individual studies as well as Meta-analysis estimates based on 6 different configurations.

| Studies | Reference | A | C | E |
| --- | --- | --- | --- | --- |
| Study 3 | Steffenburg et al., 1989 | .99 (.54/.99) | .00 (.00/.46) | .01 (.00/.05) |
| Study 5 | Le Couteur et al., 1996 | .99 (.49/.99) | .00 (.00/.51) | .01 (.00/.01) |
| Study 6_1 | Taniai et al., 2008, prev 2% | .33 (.12/.71) | .67 (.29/.88) | .00 (.00/.01) |
| Study 6_2 | Taniai et al., 2008, prev 5% | .44 (.17/.92) | .56 (.08/.83) | .00 (.00/.01) |
| Study 8 | Lichtenstein et al., 2010* | .79 (.29/.92) | .03 (.00/.44) | .18 (.08/.36) |
| Study 9_1 | Hallmayer et al., 2011, prev 0.6% | .59 (.40/.82) | .38 (.15/.56) | .03 (.01/.06) |
| Study 9_2 | Hallmayer et al., 2011, prev 5% | .95 (.68/.98) | .00 (.00/.26) | .05 (.02/.12) |
| Study 12 | Nordenbaek et al., 2014, prev 5% | .99 (.69/.1.00) | .00 (.00/.31) | .00 (.00/.00) |
| Study 13 | Colvert, Tick et al., 2015 | .79 (.56/.98) | .19 (.00/.43) | .01 (.00/.05) |
|  |  |  |  |  |
| Study  3, 5, 8, 12, 13,  6_1, & 9_1 | Meta-analysis, using reported prevalence as fixed TH (TH in St8 estimated) | .74 (.70/.87) | .25 (.12/.37) | .01 (.01/.03) |
| Study  3, 5, 8, 12, 13,  6_2, & 9_2 | Meta-analysis, changing prevalence of ASD to 5% in St6 & St9 (TH in St8 estimated) | .93 (.77/.99) | .06 (.00/.21) | .01 (.01/.03) |
|  |  |  |  |  |
| Study  5, 8, 12, 13  6_1 & 9_1 | Meta-analysis, studies after 1995 using broader phenotype, using reported prevalence as fixed TH (TH in St8 estimated) | .72 (.60/.86) | .26 (.13/.39) | .02 (.01/.03) |
| Study  5, 6, 8, 9, 12, 13 | Meta-analysis, studies after 1995 using broader phenotype, using prevalence of **5% for all** (TH st8 estimated) | .91 (.76/.99) | .07 (.00/.22) | .02 (.01/.04) |
| Study  5, 6, 8, 9, 12, 13 | Meta-analysis, studies after 1995 using broader phenotype, using prevalence of 3**% for all** (TH St8 estimated) | .81 (.67/.96) | .18 (.03/.32) | .02 (.01/.03) |
| Study  5, 6, 8, 9, 12, 13 | Meta-analysis, studies after 1995 using broader phenotype, using prevalence of **1% for all** (TH St8 estimated) | .64 (.53/.77) | .35 (.22/.46) | .01 (.01/.02) |

* Threshold for Study 8 estimated at around 2.40 z-score, equivalent to prevalence value of 0.08%.
